# Supplementary material for: Validation of the Modified Helkimo Clinical Index for Diagnosing Temporomandibular Disorders in a Romanian Patient Sample
Source: Diagnostics (Basel). 2025 Sep 16;15(18):2347. doi: 10.3390/diagnostics15182347 (PMC12469251; doi:10.3390/diagnostics15182347)
Supplement: Supplementary file 1 [file diagnostics-15-02347-s001.zip › diagnostics-3838440-supplementary.pdf]

Section & Topic	No	Item	Reported on page #
		**Validation of the Modified Helkimo Clinical Index for Diagnosing Temporomandibular Disorders in a Romanian Patient Sample**	
**TITLE OR ABSTRACT**			
	**1**	The title and abstract explicitly state the study's purpose is to validate a diagnostic index and provide measures of accuracy, including sensitivity, specificity, and AUC.	1
**ABSTRACT**			
	**2**	The abstract is structured with clear headings for Background and Objectives, Methods, Results, and Conclusions.	1
**INTRODUCTION**			*2,3*
	**3**	The background highlights the challenge of diagnosing TMDs with the time-intensive DC/TMD protocol, positioning the modified Helkimo Index (mHI) as a potential triage test to simplify and streamline the diagnostic process.	2
	**4**	The objective was to assess the diagnostic validity and reliability of the mHI in a Romanian patient cohort by comparing it to the gold-standard DC/TMD protocol. The study also aimed to quantify the application time.	2,3
**METHODS**			
Study design	**5**	This was a cross-sectional study where data were collected from participants at a single point in time. The study was planned before data collection, making it prospective in its design.	3
Participants	**6**	Inclusion criteria: Patients with a confirmed TMD diagnosis based on the DC/TMD protocol for the TMD group, and individuals without TMD for the control group.<br><br>Exclusion criteria: Patients with significant systemic conditions or those who refused to participate.	3,4
	**7**	Participants were identified based on a clinical TMD diagnosis using the DC/TMD protocol for the patient group and on the absence of TMD symptoms for the control group.	3,4
	**8**	Participants were recruited at the Department of Pediatric and Adult Dentistry at the County Clinical Emergency Hospital in Galați, Romania, from January to April 2025.	3
	**9**	The participants were part of a convenience series from the population attending the clinic, rather than a random or consecutive sample.	3
Test methods	**10a**	The modified Helkimo Index (mHI) was evaluated through clinical measurements of the temporomandibular joint and masticatory muscles, assessing parameters like range of motion, muscle and joint pain on palpation, and joint sounds.	4-6
	**10b**	The Diagnostic Criteria for Temporomandibular Disorders (DC/TMD) protocol served as the gold standard. This includes both a clinical examination (Axis I) and a psychosocial assessment (Axis II).	4-7
	**11**	The DC/TMD protocol was chosen as the reference standard because it is the internationally recognized gold standard for diagnosing TMDs, providing a comprehensive and validated framework.	3-7
	**12**	The mHI results were categorized into specific scores, with a pre-specified cut-off point used to distinguish between a positive (TMD) and a negative (non-TMD) diagnosis.	4,5
	**13**	A total of 212 individuals were assessed for eligibility. Of these, 164 were enrolled (82 TMD patients, 82 controls).	7
Analysis	**14**	TMD Group: Mean age 28.0 ± 6.0 years; 51% female.<br><br>Control Group: Mean age 31.0 ± 6.5 years; 56% female. Other characteristics like BMI, residence, and athletic activity were also reported and compared between the groups.	8
	**15**	The DC/TMD protocol confirmed the diagnosis in the TMD group.	8-10
	**16**	The mHI demonstrated a sensitivity of **0.86** and a specificity of **0.84**.	12
	**17**	The study showed strong agreement between the mHI and DC/TMD diagnoses (r = 0.83).	10,12
	**18**	The study's sample size calculation (n=128) ensured adequate statistical power to minimize imprecision.164 participants recruited from the Department of Pediatric and Adult Dentistry at the County Clinical Emergency Hospital in Romania between January and April 2025 were included in the study. A priori sample size calculation was conducted to ensure adequate statistical power. Based on an expected effect size of d = 0.5, α = 0.05, and power = 0.80, a minimum of 128 participants was required. Our final sample of 164 (82 per group) exceeded this	3

CC BY SA

		threshold, ensuring sufficient precision for validity and reliability analyses. Eligible participants were between 18 and 45 years of age.	
**RESULTS**			
Participants	**19**	The mean age was slightly lower in the TMD group (28.0 ± 6.0 years) compared with controls (31.0 ± 6.5 years; p = 0.02), with a moderate effect size (d = 0.49). Sex and BMI distributions were comparable across groups (p > 0.3). Interestingly, a significantly higher proportion of TMD patients reported being athletes (60% vs. 48%; p = 0.004), suggesting a possible association between sports activity and TMD occurrence. Urban–rural distribution also differed significantly (p = 0.003).	8
	**20**	Table 3. Baseline Demographic and Clinical Characteristics	7,8
	**21**	The study confirmed that the mHI is a valid, reliable, and time-efficient tool for diagnosing TMDs, directly addressing the study objectives.	
	**22**	Time interval and any clinical interventions between index test and reference standard	
Test results	**23**	Cross tabulation of the index test results (or their distribution) by the results of the reference standard	
	**24**	Estimates of diagnostic accuracy and their precision (such as 95% confidence intervals)	
	**25**	Any adverse events from performing the index test or the reference standard	
**DISCUSSION**			
	**26**	The study's limitations include a single-center design with a potentially homogenous population, which may affect the generalizability of the findings. The study also did not account for psychological factors, which are known to influence TMDs.<br>The findings suggest the mHI is a valuable tool for initial patient triage and screening in general dental practice, but further research is needed in diverse populations to confirm its broader applicability.	16,17
	**27**	Our study demonstrates that the modified Helkimo Index (mHI) is a valid, reliable, and operationally efficient clinical alternative to the DC/TMD protocol for diagnosing temporomandibular disorders. Its high performance in terms of sensitivity, specificity, and reproducibility, combined with its short administration time, supports the broad implementation of the mHI in general dental practice and resource-limited settings. These results validate the mHI as a pragmatic tool with genuine potential to optimize triage and early diagnosis of TMD in diverse clinical contexts.	17
**OTHER INFORMATION**			
	**28**	The study was conducted following the Declaration of Helsinki and approved by the Institutional Review Board (or Ethics Committee) of" Dunărea de Jos" University. (no. 16/CEU/2024, 9 December 2024).	17
	**29**	At the University Register for Ethics Committee approves studies involving human subjects.	
	**30**	The document states that there was no external funding.	

STARD 2015

AIM

STARD stands for “Standards for Reporting Diagnostic accuracy studies”. This list of items was developed to contribute to the completeness and transparency of reporting of diagnostic accuracy studies. Authors can use the list to write informative study reports. Editors and peer-reviewers can use it to evaluate whether the information has been included in manuscripts submitted for publication.

EXPLANATION

A **diagnostic accuracy study** evaluates the ability of one or more medical tests to correctly classify study participants as having a **target condition.** This can be a disease, a disease stage, response or benefit from therapy, or an event or condition in the future. A medical test can be an imaging procedure, a laboratory test, elements from history and physical examination, a combination of these, or any other method for collecting information about the current health status of a patient.

The test whose accuracy is evaluated is called **index test.** A study can evaluate the accuracy of one or more index tests. Evaluating the ability of a medical test to correctly classify patients is typically done by comparing the distribution of the index test results with those of the **reference standard**. The reference standard is the best available method for establishing the presence or absence of the target condition. An accuracy study can rely on one or more reference standards.

If test results are categorized as either positive or negative, the cross tabulation of the index test results against those of the reference standard can be used to estimate the **sensitivity** of the index test (the proportion of participants *with* the target condition who have a positive index test), and its **specificity** (the proportion *without* the target condition who have a negative index test). From this cross tabulation (sometimes referred to as the contingency or “2x2” table), several other accuracy statistics can be estimated, such as the positive and negative **predictive values** of the test. Confidence intervals around estimates of accuracy can then be calculated to quantify the statistical **precision** of the measurements.

If the index test results can take more than two values, categorization of test results as positive or negative requires a **test positivity cut-off**. When multiple such cut-offs can be defined, authors can report a receiver operating characteristic (ROC) curve which graphically represents the combination of sensitivity and specificity for each possible test positivity cut-off. The **area under the ROC curve** informs in a single numerical value about the overall diagnostic accuracy of the index test.

The **intended use** of a medical test can be diagnosis, screening, staging, monitoring, surveillance, prediction or prognosis. The **clinical role** of a test explains its position relative to existing tests in the clinical pathway. A replacement test, for example, replaces an existing test. A triage test is used before an existing test; an add-on test is used after an existing test.

Besides diagnostic accuracy, several other outcomes and statistics may be relevant in the evaluation of medical tests. Medical tests can also be used to classify patients for purposes other than diagnosis, such as staging or prognosis. The STARD list was not explicitly developed for these other outcomes, statistics, and study types, although most STARD items would still apply.

DEVELOPMENT

This STARD list was released in 2015. The 30 items were identified by an international expert group of methodologists, researchers, and editors. The guiding principle in the development of STARD was to select items that, when reported, would help readers to judge the potential for bias in the study, to appraise the applicability of the study findings and the validity of conclusions and recommendations. The list represents an update of the first version, which was published in 2003.

More information can be found on http://www.equator-network.org/reporting-guidelines/stard.

cc BY SA
